# Tumeur d’Abrikossoff à localisation gastrique: à propos d’un nouveau cas

**DOI:** 10.11604/pamj.2017.28.220.6583

**Published:** 2017-11-09

**Authors:** Youssef Hnach, Mohamed Allaoui, Mohamed Oukabli

**Affiliations:** 1 Service de Gastro-entérologie, 1er Centre Médico-chirurgical, Agadir, Maroc; 2 Service d’Anatomie Pathologique, Hopital Militaire d’Instruction Mohammed V, Rabat, Maroc

**Keywords:** Tumeurs à cellules granuleuses, résection endoscopique, tumeur d´Abrikossoff, Granular cell tumors, endoscopic resection, Abrikossoff tumor

## Abstract

Les tumeurs à cellules granuleuses (TCG) sont des tumeurs peu fréquentes. Elles sont localisées préférentiellement eu niveau de la peau et des tissus sous-cutanés. La localisation gastrique reste rare. A un nouveau cas de tumeur d'Abrikossoff à localisation gastrique ainsi qu'une brève revue de la littérature, on se propose d'étudier les particularités cliniques, endoscopiques et thérapeutiques de cette entité rare.

## Abstract (en)

Granular cell tumors (GCT) are uncommon tumors. They mainly occur at the level of the skin and the subcutaneous tissues. Gastric tumor is rare. We here report a new case of gastric Abrikossoff tumor as well as a brief literature review. This study aims to examine the clinical, endoscopic and therapeutic features of this rare disease.

## Introduction

Les tumeurs à cellules granuleuses (TCG) ont été décrites pour la première fois par Abrikosoff en 1926. Ils sont rares et le plus souvent bénignes. Ils touchent essentiellement la peau, les tissus sous-cutanés, la langue, le sein et l'appareil génital féminin. L'atteinte du tube digestif demeure rare ne dépassant pas 8% de l´ensemble (TCG) [1] et parmi ceux-ci le site le plus frequent est l'œsophage, suivie du gros intestin [2]. La localisation gastrique est exceptionnelle, de nos jours 24 cas seulement ont été décrits [3]. A travers cette nouvelle observation nous entailleront les particularités cliniques, endoscopiques, histologiques et thérapeutiques de cette entité.

## Patient et observation

**Figure 1:**
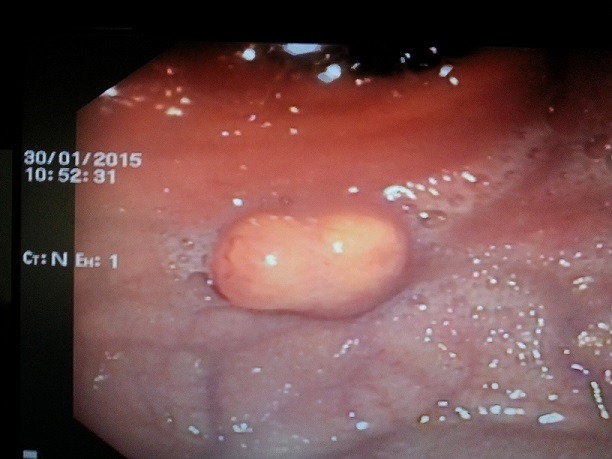
Aspect endoscopique à la fibroscopie œso-gastro-duodénale montrant une lésion bourgeonnante d’allure sous-muqueuse de la grande courbure fundique d’environ 25 mm de diameter

**Figure 2:**
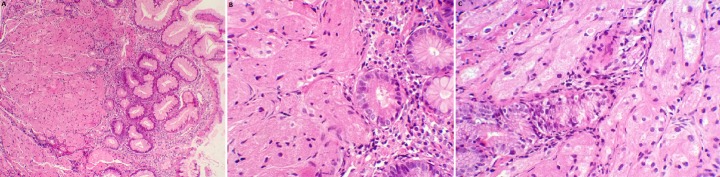
A) muqueuse gastrique siège d’une prolifération tumorale en nappes (HE, x50); B) les cellules tumorales sont de grande taille, grossièrement arrondies (HE, x400); C) elles sont pourvues d’un cytoplasme abondant éosinophile, d’aspect granulaire et de petits noyaux uniformes (HE, x400)

Une jeune patiente de 20 ans a été admise au sein de notre formation pour exploration endoscopique d'une dysphagie basse aux solides évoluant depuis 3 mois, sans antécédents pathologiques notables ni altération de l'état général, l'examen clinique est sans particularité, le bilan biologique a objectivé une anémie ferriprive à (Hb: 10g/dl). La vidéo endoscopie œso-gastro-duodénale retrouve une lésion bourgeonnante d'allure sous-muqueuse de la grande courbure fundique d'environ 25mm de diamètre, recouverte d'une muqueuse normale, sans autre anomalie (Figure 1). L'étude anatomopathologique ayant objectivée des fragments de muqueuse gastrique type fundique à chorion siège d'un processus prolifératif bénin. Il est fait de grandes cellules polyédriques à cytoplasme éosinophile, granuleux, à petits noyaux ronds hyperchromes, réguliers. Elles s'agencent en cordons réguliers séparés par un stroma peu abondant. Le diagnostic histologique de tumeur à cellules granuleuses d'Abrikossof fundique a été retenu (Figure 2). La recherche d'une autre localisation de tumeur à cellules granuleuses, notamment oto-rhino-laryngée par un examen clinique et endoscopique, cutanée par un examen cutané minutieux et pulmonaire par un scanner thoracique était négative. Le bilan d'extension comprenant une écho-endoscopique et une TDM thoraco-abdomino-pelvienne ayant conclu à une lésion touchant la muqueuse et la sous-muqueuse respectant la musculeuse sans adénopathies de voisinage ni métastases à distance. La résection endoscopique par mucosectomie a été réalisée sans incidents, deux clips hémostatiques a visée prophylactique ont été placés. Une surveillance endoscopique est prévue à 3 mois.

## Discussion

Les tumeurs à cellules granuleuses (TCG) ont été décrites en 1926 par Abrikossoff qui a rapporté une série de cinq cas de tumeurs bénignes au niveau de la langue, alors qualifiées de « myoblastome à cellules granuleuses » [4]. Ces tumeurs sont rares (0,019% à 0,03% toutes tumeurs confondues), siègent essentiellement au niveau de la tête et du cou avec une atteinte préférentielle de la peau, du tissu sous cutané et de la langue [5]. La localisation digestive demeure exceptionnelle ne dépassant pas 5% et la localisation œsophagienne est la plus fréquente [6]. La localisation gastrique est associée à une localisation œsophagienne Synchrone dans 50% des cas [7]. Il s'agit le plus souvent de tumeurs sous-muqueuses, allant de quelques millimètres a plusieurs centimètres, Les TGC sont le plus souvent bénignes, quelques cas de malignité ont été décrits [8]. L'histogenèse des TCG demeure incertaine, l'origine neurogène est de nos jours l'hypothèse la plus admise du fait de l'immunomarquageneurogène (NSE et PS100) et de l'existence de neurofilaments à l'étude ultrastucturale [9]. Les biopsies doivent être multiples et profondes, permettent le diagnostic dans 80% des cas. Histologiquement, la tumeur comme c'est le cas de notre patiente est caractérisée par une prolifération de cellules de grandes tailles, polygonales, séparées par un stroma grêle avec un cytoplasme abondant chargé de fines granulations éosinophiles, les noyaux sont hyperchromatiques. À l'immunohistochimie, les cellules tumorales sont presque toujours positives pour les marqueurs neurogènes: protéine S100 (100% des cas) et Neurone Specific Enolase ou NSE (90% des cas) [4]. L'échoendoscopie a permis l'étude municieuse de l'invasion tumorale de la paroi gastrique. L'exérèse endoscopique demeure le traitement de choix en absence d'envahissement de la musculeuse, autrement la résection chirurgicale semble inévitable [7].

## Conclusion

Les tumeurs d'Abrikossoff sont des tumeurs rares, exceptionnellement malignes, dont le diagnostic peut être trompeur, d'où l'intérêt des biopsies per-endoscopiques systématiques de toute lésion. Leur aspect histologique est habituellement caractéristique. Leur prise en charge n'est pas consensuelle et fait habituellement appel à la résection endoscopique en cas de petite tumeur et au traitement chirurgical pour les tumeurs de plus grande taille.

## Conflits d’intérêts

Les auteurs ne déclarent aucun conflit d'intérêts.
